# Protective HLA alleles are associated with reduced LPS levels in acute HIV infection with implications for immune activation and pathogenesis

**DOI:** 10.1371/journal.ppat.1007981

**Published:** 2019-08-26

**Authors:** Daniel T. Claiborne, Eileen P. Scully, Christine D. Palmer, Jessica L. Prince, Gladys N. Macharia, Jakub Kopycinski, Clive M. Michelo, Howard W. Wiener, Rachel Parker, Krystelle Nganou-Makamdop, Daniel Douek, Marcus Altfeld, Jill Gilmour, Matt A. Price, Jianming Tang, William Kilembe, Susan A. Allen, Eric Hunter

**Affiliations:** 1 Emory Vaccine Center, Yerkes National Primate Research Center, Emory University, Atlanta, Georgia, United States of America; 2 Ragon Institute of MGH, MIT and Harvard, Cambridge, Massachusetts, United States of America; 3 Human Immunology Laboratory, International AIDS Vaccine Initiative, London, United Kingdom; 4 Zambia-Emory HIV Research Project, Lusaka, Zambia; 5 Department of Epidemiology, University of Alabama at Birmingham, Birmingham, Alabama, United States of America; 6 Department of Pathology and Laboratory Medicine, Emory University, Atlanta, Georgia, United States of America; 7 Vaccine Research Center, National Institute of Allergy and Infectious Diseases, National Institutes of Health, Bethesda, Maryland, United States of America; 8 Virus Immunology Unit, Heinrich-Pette-Institut, Hamburg, Germany; 9 International AIDS Vaccine Initiative, New York, New York, United States of America; 10 Department of Epidemiology and Biostatistics, University of California at San Francisco, San Francisco, California, United States of America; 11 Department of Medicine, University of Alabama at Birmingham, Birmingham, Alabama, United States of America; University of Wisconsin, UNITED STATES

## Abstract

Despite extensive research on the mechanisms of HLA-mediated immune control of HIV-1 pathogenesis, it is clear that much remains to be discovered, as exemplified by protective HLA alleles like HLA-B*81 which are associated with profound protection from CD4+ T cell decline without robust control of early plasma viremia. Here, we report on additional HLA class I (B*1401, B*57, B*5801, as well as B*81), and HLA class II (DQB1*02 and DRB1*15) alleles that display discordant virological and immunological phenotypes in a Zambian early infection cohort. HLA class I alleles of this nature were also associated with enhanced immune responses to conserved epitopes in Gag. Furthermore, these HLA class I alleles were associated with reduced levels of lipopolysaccharide (LPS) in the plasma during acute infection. Elevated LPS levels measured early in infection predicted accelerated CD4+ T cell decline, as well as immune activation and exhaustion. Taken together, these data suggest novel mechanisms for HLA-mediated immune control of HIV-1 pathogenesis that do not necessarily involve significant control of early viremia and point to microbial translocation as a direct driver of HIV-1 pathogenesis rather than simply a consequence.

## Author summary

During acute HIV infection, there exists a complex interplay between the host immune response and the virus, and the balance of these interactions dramatically affects disease trajectory in infected individuals. Variations in Human Leukocyte Antigen (HLA) alleles dictate the potency of the cellular immune response to HIV, and certain well-studied alleles (HLA-B*57, B*27) are associated with control of HIV viremia. However, though plasma viral load is indicative of disease progression, the number of CD4+ T cells in the blood is a better measurement of disease severity. Through analysis of a large Zambian acute infection cohort, we identified HLA alleles that were associated with protection for CD4+ T cell loss, without dramatic affect on early plasma viremia. We further link these favorable HLA alleles to reduction in a well-known contributor to HIV pathogenesis, the presence of microbial products in the blood, which is indicative of damage to the gastrointestinal tract, a process which accelerates disease progression in HIV infected individuals. Ultimately, these results suggest a new mechanism by which the cellular immune response can combat HIV-associated pathogenesis, and further highlight the contribution of gut damage and microbial translocation to accelerating disease progression, even at early stages in HIV infection.

## Introduction

The factors underlying HIV-1 pathogenesis are a complex interplay between the host and virus. We have previously shown that viral characteristics, such as the replicative fitness of the transmitted variant, are significant predictors of early immune activation and further disease progression in HIV-1 infected individuals [1]. With respect to host factors, a series of genome-wide association studies (GWAS) have identified the HLA I locus to be the primary site of polymorphisms significantly affecting HIV-1 disease outcome [2–4]. Variations at this locus have also been linked to the efficiency of T cell control of viremia [5,6], and viremia is a strong predictor of HIV-1 disease progression [7–10]. Specific HLA class I alleles, such as B*57 and B*27, are consistently enriched in HIV-1 controllers, reinforcing the role of these genes in viral suppression [11]. However, the role of HLA may extend beyond viral control; in a multi-country HIV cohort study, the HLA class I allele, B*81, was shown to be associated with protection from CD4+ T cell decline without significant control of plasma viremia [12], suggesting that there are additional mechanisms of HLA-mediated CD4+ T cell protection distinct from early control of plasma viral load. We propose that additional immunogenetic factors with this protective phenotype exist, and furthermore, that these factors alter early events post infection, before the establishment of a viral load set point.

The most potent clinical indicator of HIV-1 pathogenesis and the severe immune depletion of AIDS is the CD4+ T cell count in the peripheral blood. CD4+ T cell decline during HIV-1 infection has been shown to be more accurately predicted by levels of chronic immune activation than viral load, underlining the importance of both host and virus in pathogenesis [13–15]. The determinants of this chronic immune activation are multiple, and include viral activity along with microbial translocation [16–18]. Microbial translocation is a major contributor to immune activation and disease progression in HIV-1 infection [19]. The early loss of gut mucosal integrity with the infection and depletion of CD4+ T cells locally is thought to facilitate the transit of bacterial components into the bloodstream [20]. Studies have linked both microbial DNA and LPS levels to T cell activation and amplification of viremia [21].

However, there are differences in baseline levels of detectable microbial translocation in specific populations, with evidence of ongoing immune activation in HIV seronegative MSM [22], suggesting that some of this pathogenesis may have host determinants. Further work has demonstrated that the composition of the microbial community in the female genital tract has a significant effect on the local inflammatory environment [23]. In addition, specific HLA alleles have been linked to particular microbial community phenotypes, and in turn to inflammatory disease susceptibility [24–27]. Taken together, there is strong evidence for the importance of microbial translocation in immune activation and HIV-1 pathogenesis, and that the composition of the microbiome is partially determined by host immunogenetics.

To elucidate novel mechanisms of immune protection, we sought to identify additional HLA alleles associated with the phenotype of CD4+ T cell preservation, irrespective of their associations with plasma viremia. This approach allows us to uncover alternate mechanisms by which the host cellular immune response alters disease trajectory following acute infection. We identified 4 HLA class I alleles and 2 HLA class II alleles significantly associated with protection from rapid CD4+ T cell decline even when correcting for early set point viral load, sex of the infected individual, and viral replicative capacity (vRC). Furthermore, the HLA class I alleles we identified were associated with a reduction in levels of circulating LPS during acute infection, and appear to be associated with CD8+ T cell responses targeting more conserved regions of Gag. Early LPS levels were a significant predictor of CD4+ T cell decline independent of plasma viral load, and were further associated with cellular immune activation, specifically in the T cell compartment. Collectively, these data suggest that microbial translocation is a driver of immune activation rather than a consequence, establishing early gut damage and microbial translocation as a potential target for therapeutic intervention at both the acute and chronic stages of HIV infection.

## Results

### HLA alleles can protect from CD4+ T cell decline without controlling early plasma viral load

To identify the contribution of protective immunogenetic factors to disease progression in a Zambian heterosexual transmission cohort, we performed HLA class I and class II typing to 4-digit resolution in a group of 127 acutely infected subjects **(S1 Table)**. Detailed clinical data was collected and plasma viral load and CD4+T cell counts measured longitudinally starting from the time of HIV-1 diagnosis (median of 44 days post estimated date of infection) and continuing at 3-month intervals thereafter up to 6 years post infection. We have previously defined viral replicative capacity (vRC) for the transmitted viruses in this cohort, as measured by an in vitro replicative fitness assay [1,28] and that data was integrated into our analysis.

We employed Cox proportional hazards models coupled with a stepwise backward variable selection approach, to identify both HLA class I and II alleles associated with protection from significant CD4+ T cell decline, which we defined as the time to a CD4+ T cell count less than 300 cells/mm^3^. Sex of the infected participant and vRC of the transmitted virus, which represent additional host and viral characteristics previously shown to significantly affect HIV-1 pathogenesis [1,29–31], were added to the model as static covariates during the backwards selection process.

In a multivariable Cox proportional hazards model, 4 HLA class I alleles (B*1401, B*57, B*5801, and B*81) and 2 HLA class II alleles (DQB1*02 and DRB1*15) were found to be independent protective factors delaying the loss of CD4+ T cells **(Table 1)**.

**Table 1:** HLA alleles associated with significant protection from CD4+ T cell decline.

	Cox Proportional Hazards Model (Time to CD4 counts <300)		
Factors Tested	HR	95% CI	*P*-value
Female	.66	.41–1.03	.07
Low vRC^a^	.35	.20–.59	<0.0001
B*1401	.17	.01–.77	.02
B*57/5801	.40	.20–.72	.002
B*81	< .01	.00–.16	< .0001
DQB1*02	.47	.28–.77	.002
DRB1*15	.46	.27–.76	.002

^a^Low viral replication capacity is defined as individuals infected with viruses falling within the lowest tercile the range tested

HR, hazard ratio as risk per unit change in regressor; CI, confidence interval; vRC, viral replication capacity

**Fig 1:**
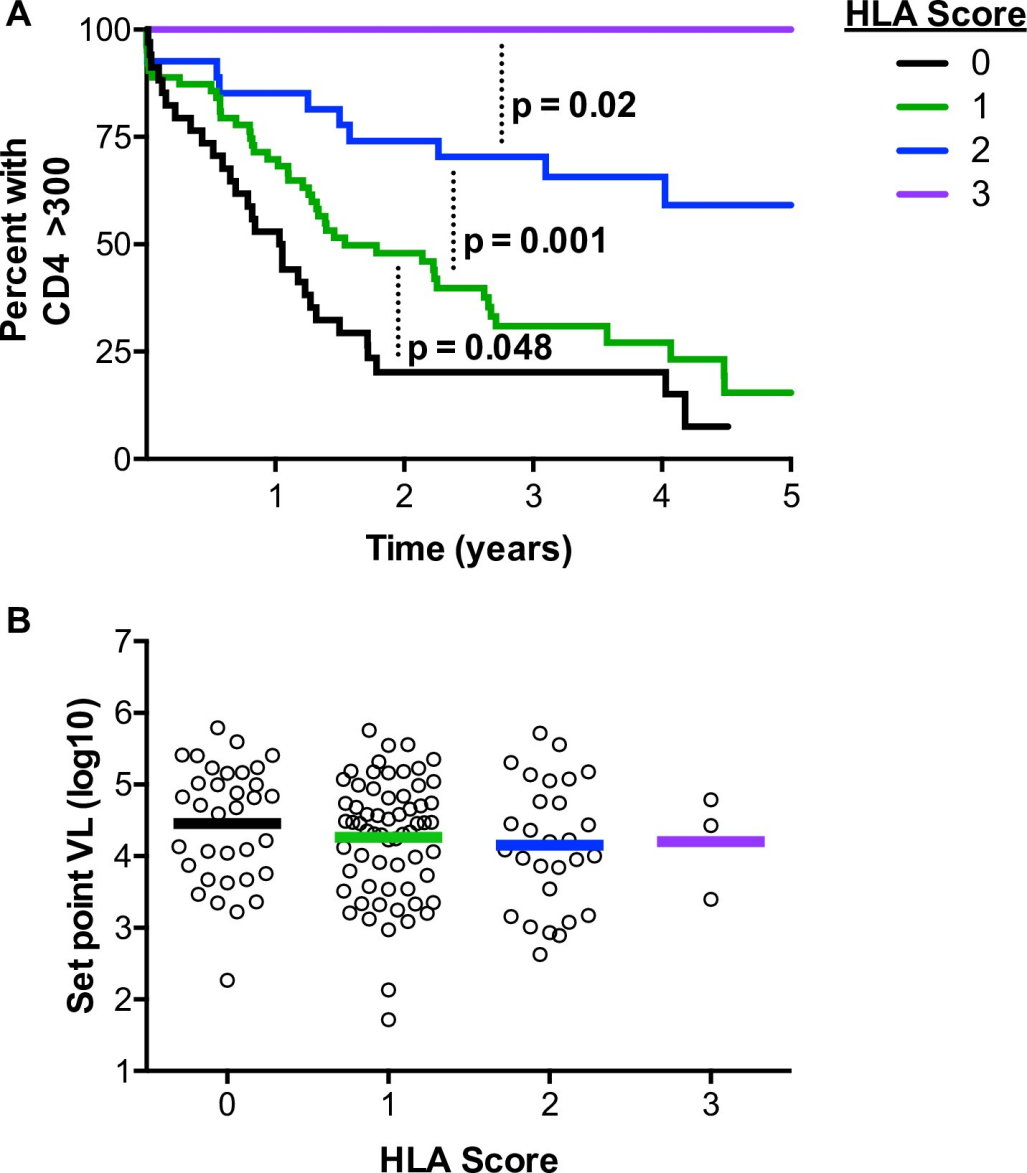
Multiplicities of protective HLA alleles and their additive effects on CD4+ T cell decline and set point viral loads. HLA-B*1401, B*57, B*5801, B*81, HLA-DQB1*02, and HLA-DRB1*15 were defined as CD4-protective in this cohort. (A) Kaplan-Meier survival curves, with an endpoint defined as CD4 counts < 300/μl, demonstrating the effect of carriage of increasing numbers of protective HLA alleles on CD4+ T cell decline. P-values represent significantly different decline trajectories between adjacent groups and were generated from a Cox proportional hazards model. (B) Mean set point viral loads between individuals carrying increasing numbers of protective HLA alleles.

As is implied by their independent predictive nature, HLA alleles affecting CD4+ T cell decline were additive in their effects and formed distinct profiles even in the absence of sex and vRC as covariates **(Fig 1A)**. Furthermore, these HLA alleles generated additive scores when divided into HLA class I or class II alleles only **(S1 Fig)**. In contrast, a significant additive effect of the protective alleles was not observed on set point viral load **(Fig 1B)**, suggesting that these alleles may affect immunopathogenesis without a substantial impact on early plasma viremia in this cohort.

To further interrogate this observation, we sought to isolate the effects from the influence of viral load. Plasma viremia, and specifically viral load set point, is a consistent and strong predictor of CD4+ T cell decline in this and multiple other HIV infection cohorts [1,32,33]. Classically, HLA alleles that are protective in the context of HIV-1 pathogenesis have been associated with significant control of plasma viremia, especially early in infection [4,34]. As the initial analyses **(Table 1)** assessed HLA alleles for their effect on CD4+ T decline without controlling for set point viral load we built a multivariable Cox proportional hazards model where set point viral load was added as a covariate. We found that all 6 HLA alleles remained significant predictors with set point viral load in the model **(S2 Table)**. Indeed, only B*57 was associated with a statistically significant decrease in early set point viral loads when analyzed in isolation **(S2 Fig)**, an association which has been described previously [35]. These data suggest that these particular HLA alleles are exerting their protective effect on CD4+ decline via an alternative mechanism and not simply through the well-described control of early plasma viremia.

### Protective HLA class I alleles are associated with a reduction in markers of gut damage in early HIV infection

**Fig 2:**
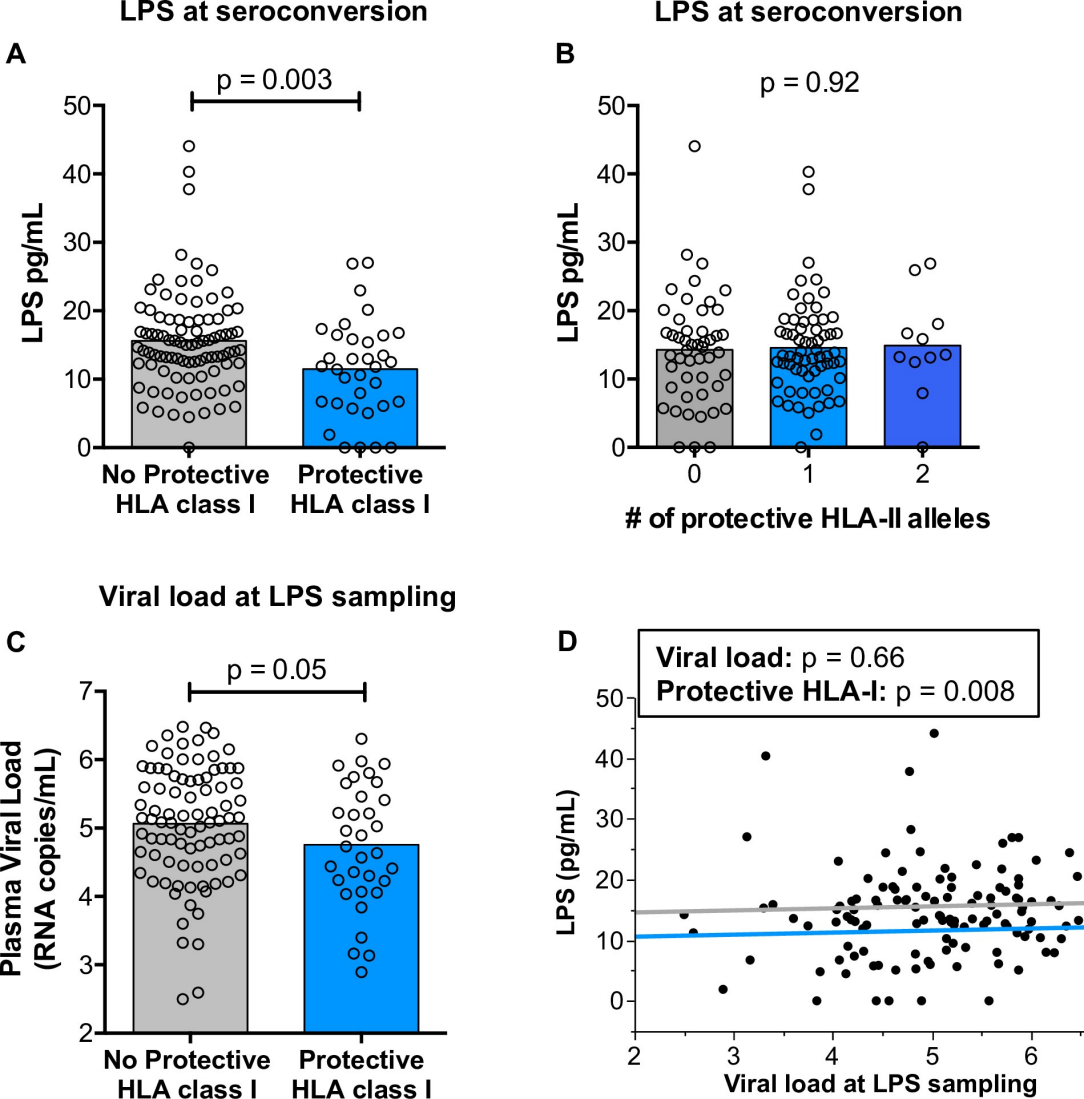
Protective HLA class I, but not HLA class II, alleles are associated with reduced levels of circulating lipopolysaccharide early after HIV infection. Lipopolysaccharide (LPS) levels were measured in the plasma for 124 seroconvertors a median of 44 days after the estimated date of infection. (A and B) The levels of LPS in the plasma were compared between individuals carrying any of the defined protective HLA class I (panel A) or HLA class II alleles (panel B). Statistical comparisons were made using the Student’s *t* test (A and C) or an ANOVA (B) and reported p-values are one-tailed. (C) Comparison of viral load at the time of LPS measurement between individuals with or without protective HLA class I alleles. (D) A graphical depiction of a generalized linear model showing the relative associations between both plasma viral load and the presence of protective HLA class I alleles with LPS levels measured in the plasma. The distance between horizontal lines represent the difference in means between individuals with (light blue) and without (gray) protective HLA class I alleles whereas the direction of the lines represents regression between the two continuous variables: viral load (x-axis) and LPS in the plasma (y-axis).

Inflammation is an alternative driver of disease progression, independent of viral load [36], and is frequently associated with gut damage and bacterial translocation [37]. We therefore investigated associations between the protective HLA alleles identified in this study and inflammatory markers linked to pathogenesis. We found a striking association between the identified protective HLA class I alleles (B*1401, B*57, B*5801, and B*81) and lower plasma LPS levels at the earliest time of sampling (median of 44 days post estimated date of infection; **Fig 2A**). This difference in LPS levels was specific for class I alleles; we observed no association between protective HLA class II alleles and circulating LPS **(Fig 2B)**. The grouped protective class I alleles were also associated with a moderate 0.31 log reduction in plasma viral loads at the time of LPS sampling **(Fig 2C)**. However, in a generalized linear model, carriage of protective class I alleles is a significant predictor of LPS levels at seroconversion (p = 0.008), whereas plasma viral loads are not (p = 0.66) **(Fig 2D)**.

**Fig 3:**
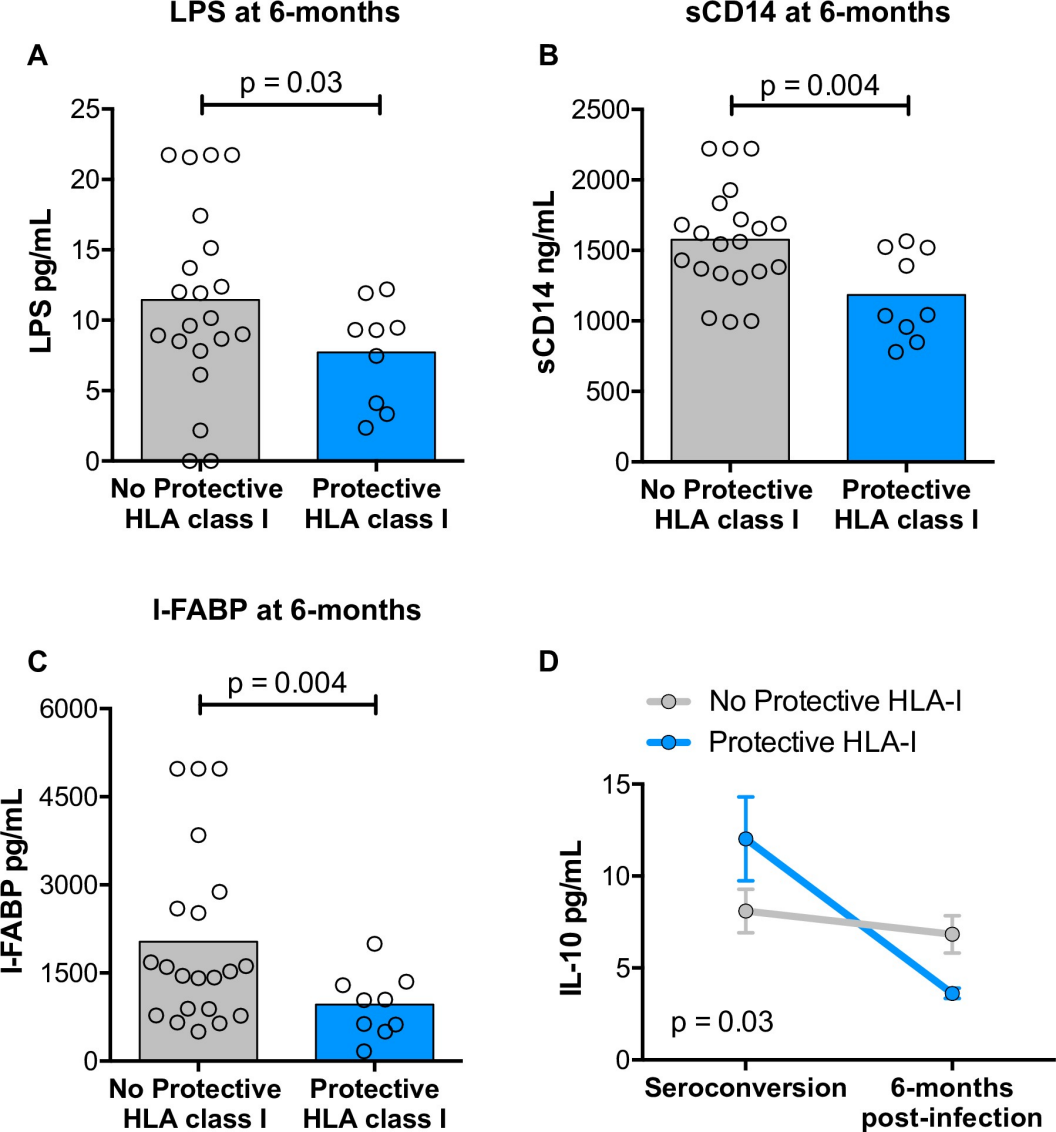
Associations between protective HLA-I alleles and markers of gut damage and microbial translocation at 6 months post HIV infection. (A–C) Comparison of levels of lipopolysaccharide, soluble CD14, and intestinal fatty acid binding protein (I-FABP) measured at 6 months post HIV infection between those with and without protective HLA-I alleles for a subset of the cohort (n = 30). Statistical comparisons were made using the Student’s *t* test and reported p-values are one-tailed. (D) Changes in IL-10 levels as measured in the plasma from seroconversion (mean of 44 days post infection) to 6 months post infection between individuals with (light blue) and without (gray) protective HLA-I alleles. Statistical comparison was made using a multivariate analysis of variance (MANOVA). Bars represent the standard error of the mean.

Protective HLA class I alleles were also associated with reduced levels of LPS, soluble CD14, and intestinal fatty acid-binding protein (I-FABP) at 6 months post seroconversion **(Fig 3A–3C)** in a subset of individuals with samples available for analysis (n = 30). These data indicate that the LPS association is durable and is reinforced by other markers of gut integrity and microbial translocation. Furthermore, individuals carrying these protective HLA-I alleles, but not those lacking them, demonstrated a significant reduction in IL-10 levels over time **(Fig 3D)**, consistent with published literature linking the presence of circulating microbial products with increased production of IL-10 by monocytes [38,39].

Other factors such as excessive alcohol use can significantly affect gut integrity and bacterial dysbiosis leading to increased levels of microbial translocation [40–42]. In this cohort, data on alcohol consumption that was previously collected [43,44] was used to generate scores identifying individuals who consumed alcohol excessively and those that did not. We observed a significant elevation in plasma LPS at seroconversion in individuals that consumed alcohol excessively **(S3 Fig)**. Importantly, in a generalized linear model, excessive alcohol consumption and protective HLA class I alleles were independent predictors of plasma LPS levels near seroconversion **(S3 Fig)**, suggesting the association between HLA class I and LPS is not significantly confounded by alcohol consumption in this cohort.

The MHC gene locus contains additional genes that play key roles in the innate and adaptive immune systems[45]. Many of the polymorphisms in this region are in strong linkage disequilibrium (LD) [46], raising the possibility that the observed associations between these 4 HLA class I alleles and microbial translocation may be attributed to other genes in this locus. To rule out this possibility, we analyzed high resolution ImmunoChip data to look for other polymorphisms in the MHC locus in strong LD with these four HLA haplotypes. We observed no SNPs with r^2^ values >0.8 **(S4 Fig)**. This suggests that the observed reduction in microbial translocation is likely attributable to the presence of these 4 protective HLA class I alleles and not other immune-related genes in the MHC locus.

### Lower levels of plasma LPS in acute HIV infection are directly associated with protection from early CD4+ T cell decline

**Fig 4:**
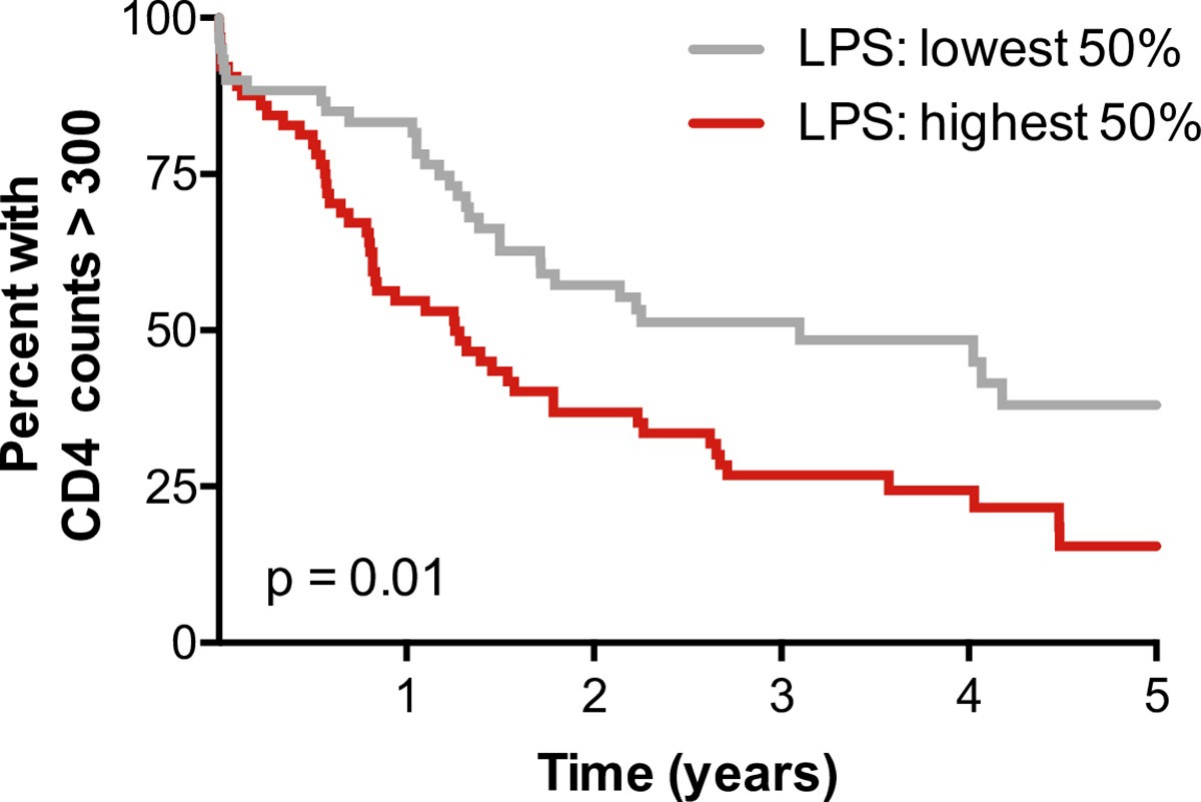
Circulating LPS levels measured during acute infection are associated with longitudinal CD4+ T cell decline. A Kaplan-Meier survival analysis comparing CD4+ T cell decline between individuals with LPS levels above or below the median for the cohort (n = 124). Thirteen individuals with CD4-protective HLA class I alleles were contained in the >50% LPS group, and 21 in the <50% LPS group. Statistics were generated from the log-rank test.

We next assessed whether a direct relationship between LPS levels and CD4+ T cell decline existed in this cohort. While translocation of microbial products, such as LPS, has been linked to immune activation and disease progression during chronic HIV-1 infection [19], we sought to specifically establish a link between very early microbial translocation and subsequent CD4+ T cell loss. In a Kaplan-Meier survival analysis with an endpoint defined as CD4+ T cell counts falling below 300 cells/mm^3^, individuals in the lowest 50^th^ percentile of LPS levels at seroconversion exhibited significantly slower CD4+ T cell decline **(Fig 4)**. This association was independent of viral load at the time of LPS sampling **(S3 Table)**. Thus, this measure of microbial translocation early during HIV-1 infection is directly associated with the kinetics of longitudinal CD4+ T cell decline.

### Systemic LPS early in infection is associated with deleterious T cell phenotypes

In chronic HIV-1 infection, systemic LPS levels are correlated with increased cellular immune activation, specifically with an increased frequency of activated CD38+/HLA-DR+ CD8 T cells [19]. The mechanisms of this effect may include both direct stimulation of T cells by microbial products [47], as well as secondary effects mediated by innate recognition and inflammatory cytokine production [22,48].

**Fig 5:**
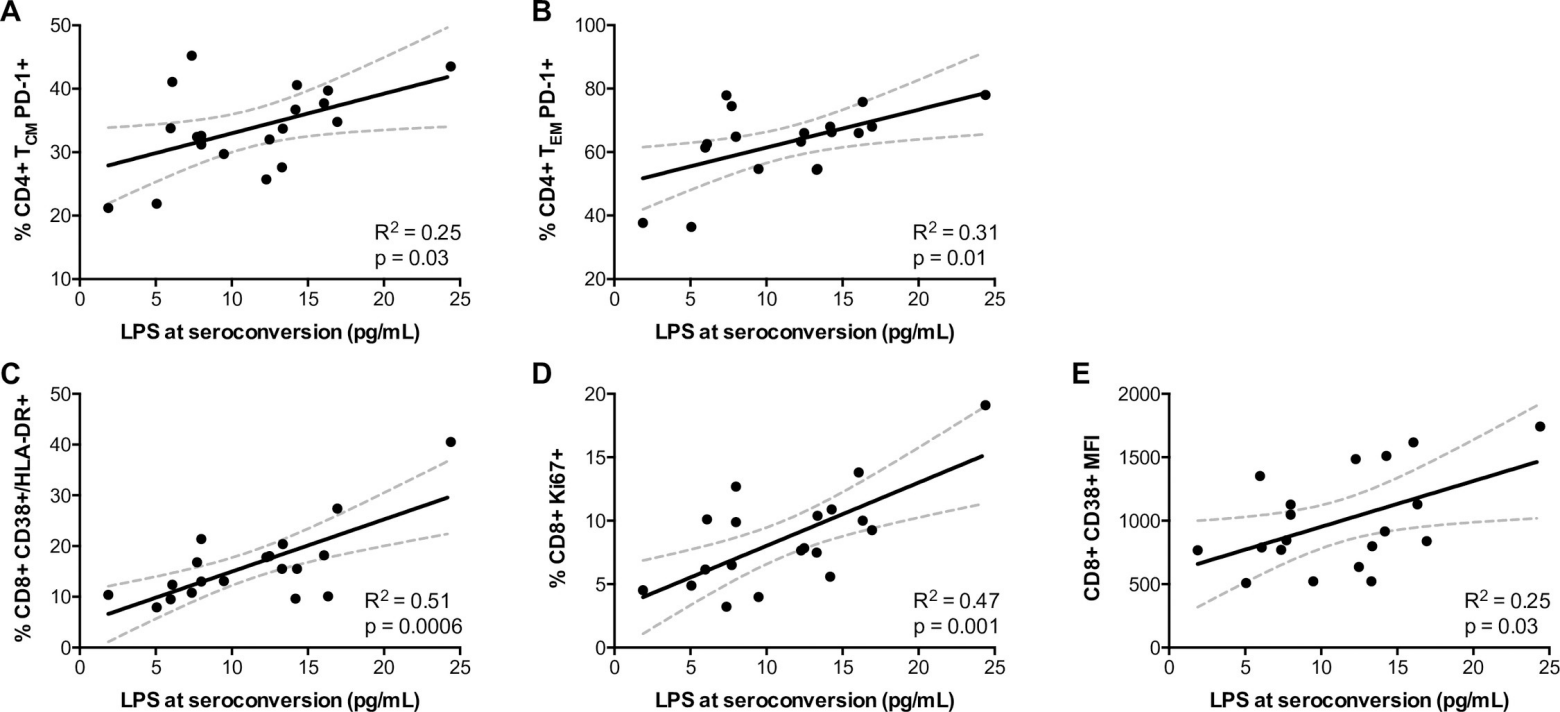
The levels of circulating LPS at seroconversion predict levels of cellular immune activation in the T cell compartment at 3 months post infection. Cryopreserved PBMCs isolated 3 months (mean of 97 days) post infection were stained for markers of immune activation, exhaustion, and cellular turnover as well as for the delineation of memory subsets in CD4 and CD8 T cells for a subset of the cohort for whom cryopreserved PBMCs were available (n = 19). Five of these 19 individuals carried CD4-protective HLA class I alleles. (A–B) Direct associations between LPS levels and the percentage of PD-1+ cells in central memory (CD45RO+/CCR7+) and effector memory (CD45RO+/CCR7-) CD4+ T cells. (C–E) Direct associations between LPS levels and markers of activation (CD38+/HLA-DR+, CD38 MFI) and turnover (Ki67+) in CD8+ T cells. Correlation statistics were generated using linear regression. Solid lines indicate trend lines and dashed lines represent 95% confidence bands.

In order to determine the effect of early microbial translocation on subsequent T cell activation in this cohort, cryopreserved PBMCs available from a subset of individuals collected 3 months after the estimated date of infection (median 97 days post estimated date of infection and median 65 days post systemic LPS measurements) were assessed by flow cytometry for markers of T cell activation and antigen experience/exhaustion. We found that LPS levels at seroconversion directly correlated with PD-1 expression on central memory and effector memory CD4+ T cells an average of two months later **(Fig 5A and 5B)**. Similarly, in the CD8+ T cell compartment, increased LPS in the periphery also correlated with the percentage of CD38 and HLA-DR double positive cells, increased percentages of Ki67+ cells, and CD38 expression levels (assessed by MFI) **(Fig 5C–5E)**. Furthermore, the associations between LPS and these activated T cell phenotypes were independent of viral loads at the time of PBMC collection **(S4 Table)**, providing further evidence for early microbial translocation as an independent predictor of immune activation and a potential driver of immunopathogenesis.

### Individuals with protective HLA class I alleles more efficiently target conserved regions of Gag

**Fig 6:**
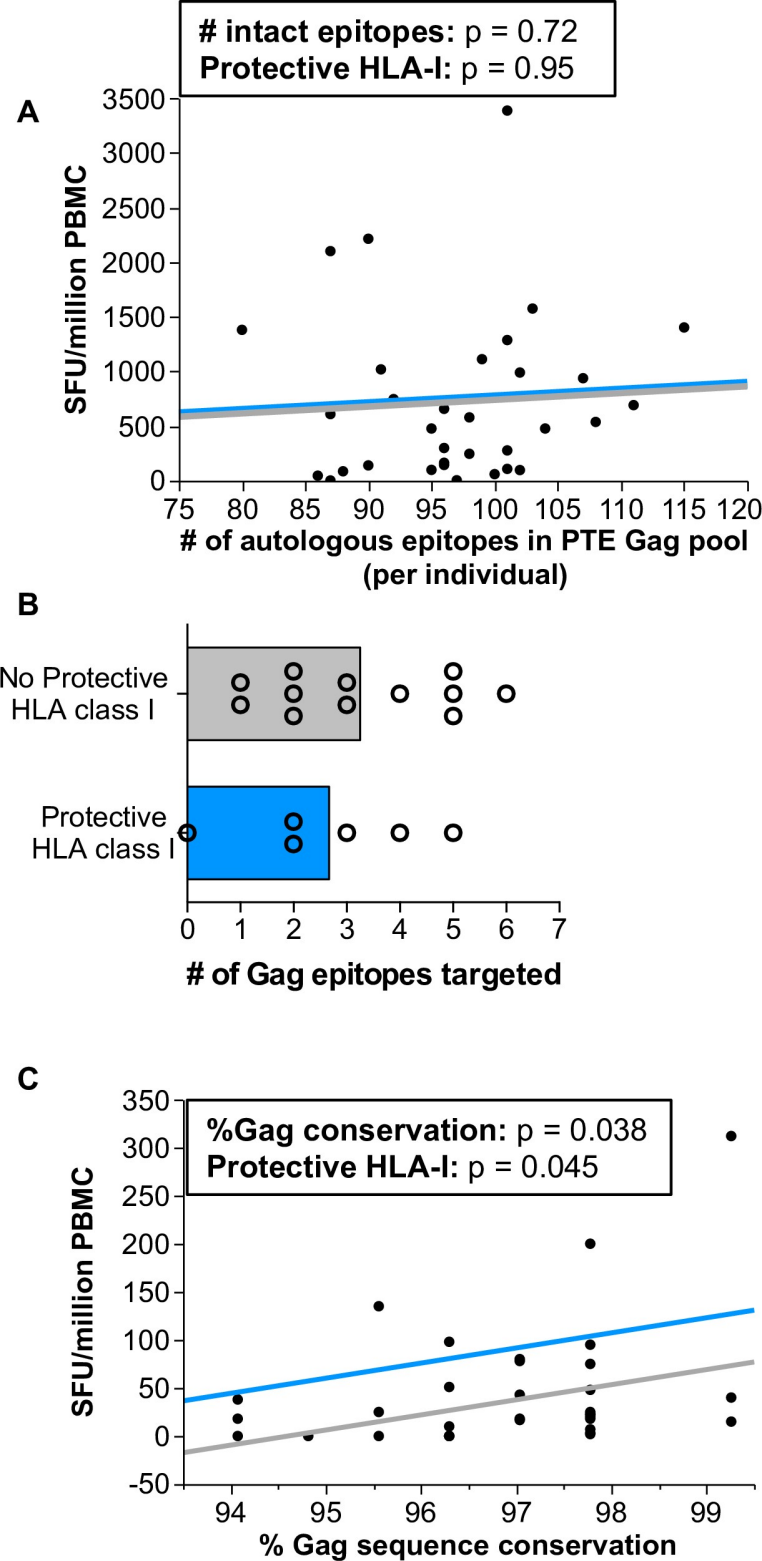
Individuals with CD4-protective HLA class I alleles more efficiently target conserved regions of Gag. (A) A graphical depiction of a generalized linear model showing the relative association between the presence of CD4-protective HLA class I alleles with IFNγ ELISpot responses to Gag PTE peptide pools from cryopreserved PBMCs collected a median of 45 days post infection. A correction factor for transmitted Gag sequence similarity to the PTE peptide pools was added as a covariate. The distance between horizontal lines represent the difference in means between individuals with (light blue, n = 6) and without (gray, n = 27) protective HLA class I alleles whereas the direction of the lines represents regression between the two continuous variables: Gag sequence similarity to peptide pools (x-axis) and IFNγ ELISpot responses reported as spot forming units per million PBMCs (y-axis). (B) Cryopreserved PBMCs collected at one-year post infection (median 339 days post infection) were interrogated with peptide pools containing 300 clade C consensus 10-mer PTE Gag peptides, with subsequent deconvolution to identify single peptide responses. The number of individual Gag peptide responses for individuals with (blue bar, n = 6) and without (gray bar, n = 12) CD4-protective HLA class I alleles are depicted. (C) A graphical depiction of a generalized linear model showing the relative association between the presence of CD4-protective HLA class I alleles with IFNγ ELISpot responses to Gag HIVconsv peptides [51] from cryopreserved PBMCs collected a median of 45 days post infection. A correction factor for transmitted Gag sequence similarity to the 3 Gag regions covered by the 31 HIVconsv Gag peptides was added as a covariate. The distance between horizontal lines represent the difference in means between individuals with (light blue, n = 6) and without (gray, n = 27) protective HLA class I alleles whereas the direction of the lines represents regression between the two continuous variables: Gag sequence similarity to HIVconsv Gag peptides (x-axis) and IFNγ ELISpot responses reported as spot forming units per million PBMCs (y-axis).

In order to gain insight regarding the quality of the CD8+ T cell response restricted by these protective HLA class I alleles, we analyzed IFNγ ELISpot data for a subset of individuals in this cohort for which early cryopreserved PMBCs were available. When PBMCs were interrogated with potential T cell epitope (PTE) Gag pools [49,50], we observed no difference in the magnitude of responses between those with or without protective HLA class I alleles, even when controlling for the relatedness of the transmitted gag sequence to the peptides contained in the PTE Gag pools **(Fig 6A)**. Furthermore, we observed no differences in the breadth of Gag targeting when analyzing the number of specific epitopes targeted between HLA-I groups **(Fig 6B)**. However, when peptide pools were optimized for sequence conservation, we observed that individuals with the protective HLA class I alleles identified in this study exhibited significantly higher IFNγ ELISpot responses to a pool of highly conserved Gag peptides **(Fig 6C)**[51–53]. Targeting more conserved regions of Gag, which are unlikely to rapidly escape due to fitness constraints [54–58], may help to explain the sustained protective effect of these alleles with the respect to longitudinal CD4+ T cell preservation.

## Discussion

In this study, we have utilized detailed clinical, virologic, and immunogenetic data from a well-defined heterosexual transmission cohort [1] to identify protective HLA alleles associated with attenuated CD4+ T cell decline irrespective of reduced early set point viral loads. Selected HLA class I (HLA-I) alleles were associated with significantly lower levels of systemic LPS during the early stages of infection, suggesting that this may be an unrecognized mechanism of protection associated with cellular immune responses restricted by favorable HLA alleles. This association was durable throughout the early stages of infection, as protective HLA class I alleles were favorably associated with additional markers of gut integrity and microbial translocation at subsequent time points.

The discovery of HLA alleles associated with protection from rapid CD4+ T cell decline without significant control of early plasma viremia is not without precedent. HLA-B*81 has been shown to be highly protective in this and in other African cohorts without significant control of early set point viral loads [12]. The importance of set point viral loads not withstanding [32,33], the study by Prentice et al., as well as the current study, serve to highlight other critical determinants of longitudinal pathogenesis as measured by CD4+ T cell counts. We have previously demonstrated that higher viral replication capacity was linked with increased inflammation and immune activation early in infection, setting the scene for subsequent pathogenesis [1]. We concluded that elevated immune activation, linked to transmission of viruses with higher replication capacity, was responsible for more rapid CD4+ T cell decline. In this present study, we observe a similar phenomenon, where the identified HLA-I alleles were protective with respect to CD4+ T cell decline independent of early set point viral loads and viral replicative capacity. These alleles were also associated with lower levels of microbial translocation as measured by systemic LPS. This leads us to hypothesize that these protective alleles may be exerting their effects of preserving CD4+ T cell counts by limiting early immune activation, perhaps by controlling in situ viral replication in key tissues, such as the gastrointestinal tract, a site of early HIV replication post transmission [59–61].

In contrast to the class I alleles identified in this study, the protective HLA class II alleles (DQB1*02 and DRB1*15) were not associated with reduced levels of LPS in early infection. The protective mechanism of HIV-specific CD4+ T cells is less clear than CD8+ T cells, especially since HIV-specific CD4+ T cells are preferentially infected and depleted by HIV during infection [62]. However, Ranasinghe et al. have previously identified one of these same alleles, DRB1*15, as significantly associated with control of plasma viremia in a subtype B infected cohort [63]. In that study, DRB1*15 alleles were shown to present a greater range of HIV-derived peptides, suggesting that these class II alleles may prime broader CD4+ T cell responses against HIV. It is not clear why we did not observe a similar association between DRB1*15 and control of viremia, but it is most likely due to the distinct nature of the cohorts studied (subtype B infected and of European descent compared to subtype C infected African populations) as well as differences in the 4-digit typing of the alleles identified, where DRB1*1503 was the most common variant in this Zambian cohort and DRB1*1502 was most prevalent in the cohort studied by Ranasinghe et al.

Translocation of bacterial-derived products into the systemic circulation was first shown to be associated with pathogenesis in HIV-1 infection by Brenchley et al., specifically in the chronic stages of infection [19]. It has been proposed that specific loss of Th17 CD4+ T cells in gut-associated lymphoid tissue (GALT) contributes to structural damage seen at the mucosal barrier, allowing subsequent translocation of bioactive bacterial products into the periphery [37,64,65]. Bacterial products such as LPS have been associated with increased immune activation, a driver of HIV pathogenesis that is independent of plasma viral loads. Although depletion of total CD4+ T cells (including Th17) in the gut lamina propria occurs early during acute infection, it has been difficult to unravel the cause/effect relationship between microbial translocation and immune activation in the context of ongoing viral replication. Several in vitro and in vivo studies have attempted to understand this relationship. In vitro studies have shown that LPS alone, as well as other TLR agonists, can drive central and effector memory CD4+ T cells into cell cycle and lead to expression of activation markers on CD8+ T cells [47]. Moreover, in vivo studies of a high-risk HIV-negative MSM cohort have shed light on the specific effects of microbial translocation and systemic LPS on cellular immune activation [22,48]. In these studies, HIV-negative individuals with detectable but subclinical levels of endotoxemia exhibited significantly elevated levels of proinflammatory cytokines such as TNFa, IP-10, and MCP-1, as well as significantly lower CD4/CD8 T cell ratios. Fluctuations in naturally occurring endotoxemia in the referenced HIV-negative cohort were also associated with altered CD4+ T cell proliferation profiles and altered monocyte function. Specifically, elevated LPS was associated with a reduced proliferative capacity in the CD4+ T cell compartment, which has clear implications for CD4+ T cell decline in HIV infection.

It is not known if the findings presented in the current study can be generalized to other populations, such as MSM, living with HIV. Innate immune cell persistence in the gut is linked to the composition of the microbiome [66] and specific microbiota features have been shown to enhance inflammation [67] underlining the significance of gut barrier maintenance and a healthy microbiome. Moreover, there is evidence that both sex and sexual preferences can influence the composition of the microbiome, leaving open questions about the intersection between gut dysbiosis, HIV infection, and systemic inflammation[68–71]. Nevertheless, in our study cohort of both men and women, the findings linking LPS to outcomes were independent of the sex of the participants, suggesting a more generalized phenomenon potentially linked to inflammation.

We demonstrate in this current study that LPS levels in the plasma measured at or near seroconversion predict longitudinal CD4+ T cell decline, with individuals in the lowest 50^th^ percentile of LPS values protected from rapid loss of CD4+ T cells. In addition, we observed correlations between circulating LPS levels at seroconversion and markers of T cell activation (CD38 MFI and CD38/HLA-DR co-expression), antigen experience/exhaustion (PD-1 expression, specifically on CD4+ memory T cells), and proliferation (Ki67 expression) at a subsequent 3-month time point, suggesting a causal link between circulating LPS and immune activation, which itself is strongly correlated with disease progression irrespective of viral loads [36]. The associations between circulating LPS and cellular immune activation are largely unaffected by the inclusion of plasma viral load at the time of PBMC sampling in a multivariable general linearized model. These data suggest that, at some of the earliest time points post infection, LPS is a driver of immune activation and establishes LPS as a predictor of CD4+ T cell decline that is independent of plasma viremia.

Interestingly, we did not observed any difference between individuals in terms of magnitude or breadth of epitope recognition as measure by IFNγ ELISpot response between those that expressed or lacked CD4-protective HLA class I alleles when using broad potential T cell epitope (PTE) Gag pools [49,50]. However, we did observe a more robust response to conserved regions of the Gag protein [51,52] in individuals expressing CD4-protective HLA-I alleles. This data demonstrates that the quality of the cytotoxic T lymphocyte response restricted by these HLA-I alleles may be responsible for their efficacy. Collectively, these data suggest that CD4-protective HLA-I alleles, perhaps through their ability to target biologically relevant epitopes, can initiate cellular immune responses capable of maintaining gut integrity and limiting the translocation of microbial products into the systemic circulation. This is possibly via robust control of viral replication at specific tissue sites, at a time when we do not observe significant control of plasma viremia. This hypothesis is supported by a study by Altfeld et al., where the authors observed a striking disconnect between cytotoxic T lymphocyte (CTL) responses measured in the peripheral blood and responses measured in the lymph nodes after structured treatment interruptions [72]. Indeed, a subset of the measured CTL responses were found exclusively in the lymph node, suggesting the cellular immune response can be compartmentalized. In addition, the influence of HLA genotype on the composition of the gut microbiome may contribute; with specific HLA types supporting the growth of more favorable microbial communities [25].

In summary, these data definitively link the presence of microbial products in the circulation during acute/early infection with subsequent CD4+ T cell decline and cellular immune activation. The levels of bacterial products found in the periphery, as measured by plasma LPS levels are modulated by protective HLA class I alleles at both seroconversion and 6 months post infection. These findings demonstrate an unappreciated mechanism of protection by favorable HLA alleles, beyond simply controlling systemic viral load, and further establish microbial translocation as a driver of HIV pathogenesis. Moreover, they offer additional supporting data that efforts to ameliorate gut damage and dysbiosis, and the subsequent immune activation this dysregulation initiates in HIV-infected patients, will have positive impacts on disease outcome.

## Methods

### Ethics statement

All participants in the Zambia Emory HIV Research Project (ZEHRP) discordant couples cohort in Lusaka, Zambia were adults at the time of participation and enrolled in human subjects protocols approved by both the University of Zambia Research Ethics Committee and the Emory University Institutional Review Board. Prior to enrollment, individuals received counseling and signed a written informed consent form agreeing to participate.

### Study subjects

The subjects included in this study were selected from the ZEHRP cohort based on being recently infected with HIV-1. All subjects were initially seronegative partners within serodiscordant cohabitating heterosexual couples, who knew their HIV status and were provided counseling and condoms, but subsequently seroconverted. All subjects were antiretroviral therapy naïve and were identified a median of 44 (IQR = 33–49) days after the estimated date of infection (EDI). The algorithm used to determine the EDI has been previously described [73]. Additional demographic and cohort data can be found in S1 Table. All subjects were infected by HIV-1 subtype C viruses.

### Viral loads and CD4 count measurements

Early set point viral load (VL) for newly infected individuals was defined as the earliest stable nadir VL value measured between 3 and 9 months post infection and which did not show a significant increase in value within a 3–4 month window. HIV plasma VL was determined at the Emory Center for AIDS Research Virology Core Laboratory using the Amplicor HIV-1 Monitor Test (version 1.5; Roche). CD4+ T cell counts were based on T-cell immunophenotyping, with assays done using the FACScount System (Beckman Coulter Ltd., London, United Kingdom) in collaboration with the International AIDS Vaccine Initiative.

### Genotyping

Genomic DNA was extracted from whole blood or buffy coats (QIAamp blood kit; Qiagen). HLA genotyping relied on a combination of PCR-based techniques, involving sequence-specific primers (Invitrogen) and sequence-specific oligonucleotide probes (Innogenetics), as described previously [74]. Ambiguities were resolved by direct sequencing of three exons in each gene, using kits (Abbott Molecular, Inc.) designed for capillary electrophoresis and the ABI 3130*xl* DNA Analyzer (Applied Biosystems).

SNP genotyping with the Illumina ImmunoChip was processed at a genomics core facility (University of Alabama at Birmingham); SNP alleles were inferred using the joint calling and haplotype phasing algorithm implemented in BEAGLECALL[75]. We completed a series of data cleaning and quality control procedures for SNPs in the xMHC region, excluding SNPs based on the following criteria: (i) duplication, (ii) missingness (call rate<98.5%), (iii) minor allele frequency <0.025 in SCs and <0.015 in SPs and (iv) deviation from Hardy–Weinberg equilibrium (P<10^−6^). Data processing and quality control procedures have been described previously for this data set[46].

### Generation of Gag-MJ4 chimeras and in vitro replication assay

Gag-MJ4 chimeras were generated from frozen plasma isolated at the seroconversion time point for 127 subjects as previously described[1]. Briefly, viral RNA was extracted from 140 ul of plasma using the Qiagen Viral RNA extraction kit (Qiagen). Combined RT-PCR and first round PCR were performed in a single reaction, and *gag* genes were amplified using a nested second round PCR. Patient-derived *gag* genes were joined with the MJ4 long terminal repeat portion via splice-overlap-extension PCR, and *gag*-LTR amplicons were cloned into the MJ4 proviral vector using NgoMIV and BclI endonuclease restriction enzymes.

The in vitro replication assay used to generate viral replication capacity (vRC) values has been described extensively [76]. Briefly, replication competent virus was made via transfection of 293T cells (American Type Culture Collection). Virus supernatants were harvested, titered on the TZM-bl indicator cell line, and used to infect the GXR25 T cell line (gift from Dr. Mark Brockman, Simon Fraser University, Burnaby, BC, Canada) at a constant multiplicity of infection. Supernatants from infected cultures were harvested every 2 days, and cultures were split to maintain healthy cell confluency. The extent of viral particles released into the supernatant was quantified via a radiolabeled reverse transcriptase assay. vRC values were generated by dividing the slope of replication of each virus by the slope of replication of wild-type MJ4.

### Cox proportional hazards models to identify protective HLA and HLA scores

Cox proportional hazards models with a backwards variable selection strategy were used to identify protective HLA alleles. All HLA alleles represented by at least 3 or more individuals in the cohort were included in the initial model. Sex of the seroconvertor as well as viral replication capacity of the transmitted founder virus (based on the gag gene alone) were added as static covariates present in every iteration of the model. HLA alleles contributing least significantly to the model (highest p-value in the model) were iteratively excluded in a stepwise fashion until all variables in the model had p-values < 0.05. Linkage disequilibrium between specific HLA alleles was accounted for during the variable selection process.

HLA scores presented in Fig 1 were generated by adding the number of HLA class I (B*1401, B*57, B*5801, B*81) and HLA class II (DQB1*02, DRB1*15) alleles identified to be CD4-protectve in this cohort for each individual. In S1A Fig and S1B Fig, only CD4-protective HLA class I alleles or CD4-protective HLA class II alleles were summed, respectively. Scores of 0, 1, 2, or 3 refer to any individual having that number of CD4-protective HLA class I and/or class II alleles.

### Evaluation of plasma analytes

Measurement of LPS levels was performed using the LAL Chromogenic Endotoxin Quantification kit (American Diagnostica). Levels of sCD14 (R&D systems) were measured using a standard ELISA based assay according to the manufacturer’s instructions. Plasma levels of IL-10 were measured using MILLIPLEX Human Cytokine/Chemokine detection kit (EMD Millipore). Samples were run in duplicate with all individuals on the same plate and wells with low bead count or coefficient of variance >30% were excluded from subsequent analysis. Plates were read on the Bio-Plex 3D Suspension Array System (Bio-Rad).

Intestinal fatty acid binding protein (I-FABP) was measured using a commercially available ELISA DuoSet assay (R&D Systems, Minneapolis, MN, USA) according to the manufacturer's instructions with minor adjustments. Plasma samples were diluted to 10% in diluent from the R&D Systems soluble CD14 ELISA kit (DC140) and plates were blocked with Sigma Blocking Buffer.

In order to maintain normal distributions and to lessen the effect of outliers, extreme positive values for each analyte measured in plasma were Winsorized to the 90^th^ percentile.

### Flow cytometry

Cryopreserved PBMCs were stained for flow cytometric analysis in a manner identical to that described in [1]. The following antibodies were used to distinguish T cell populations and activation phenotypes: CD3-APC/Cy7 clone SP34-2, HLA-DR-PerCP/Cy5.5 clone G46-6, CCR5-PE clone 3A9, Ki67- AlexaFluor700 clone B56, CCR7-PE/Cy7 clone 3D12 (BD Biosciences); CD4- APC clone OKT4, CD8-BV605 clone RPA-T8, PD-1-BV421 clone EH12.2H7 (BioLegend); CD45RO-PE/Texas Red clone UCHL1, CD27-PE/Cy5 clone 1A4CD27 (Beckman Coulter); CD38-FITC clone AT-1 (Stemcell).

All flow cytometry data was collected on an LSRII (BD Biosciences) cytometer using FACSDiVa version 6.3.1 software. Analysis of the data performed using FlowJo version 9.7.5 software (TreeStar).

### Alcohol use data and classifications

Data on alcohol consumption was collected for individuals in the ZEHRP cohort as described previously [43,44]. In order to categorize individuals into those that frequently drank alcohol to excess and those that did not, we made use of data collected from questions regarding how often in the last year individuals got drunk. Initial scores were denoted as follows: 1 = never, 2 = less than monthly, 3 = monthly, 4 = weekly, 5 = daily or almost daily. For the purpose of this study, individuals with scores 2 or less were denoted as “drunk less than monthly” and individuals with scores 3 or greater were denoted as “drunk monthly or more”.

### IFNγ ELISpot assays measuring Gag-specific responses

Cryopreserved PBMCs collected at the first study visit post infection (median 45 post estimated date of infection) from 33 individuals were thawed, rested overnight, and interrogated with two PTE Gag peptide pools (HIV-1 PTE Gag Peptide Pool from NIAID, DAIDS) and a pool of 31 15-mer peptides spanning 3 highly conserved regions in Gag [51] via an IFNγ ELISpot assay as previously described [77,78]. Spot forming units per million PBMCs for the two PTE pools were summed. Correction factors for sequence conservation of the transmitted Gag amino acid sequence was calculated for each of the 33 individuals, specific for either the PTE or conserved Gag peptide pools. For the PTE pool correction factor, all 320 PTE peptides were aligned to full length Gag amino acid sequences derived from viral RNA isolated from the seroconversion time point (median of 45 days post estimated date of infection) for all 33 individuals. Scores were subsequently calculated by summing the number of PTE peptides that were at least 90% conserved with respect to the individual’s Gag sequence. For the conserved epitope pool correction factor, Gag amino acid sequences from infected individuals were aligned to the 3 distinct regions covered by this pool of 31 15-mer peptides [51]. Percent similarity to these 3 regions was calculated by extracting the pairwise identity based on the sequence alignment performed using Geneious bioinformatics software (Biomatters, Aukland, NZ). The nucleotide sequences corresponding to the amino acid sequences used for these calculations have previously been deposited under GenBank accession nos. KP715723–KP715849.

Peptide specific IFNγ ELISpot responses to measure breadth were performed on a separate subset of cryopreserved PBMCs collected a median of 339 days post infection for a total of 18 individuals in the cohort [79,80]. PBMCs were thawed and expanded ex vivo using a bi-specific CD3/CD4 antibody [81]. Expanded cells were then used to interrogate responses to a matrix of peptide pools with subsequent deconvolution to single peptides [82].

### Statistical analyses

All statistical analysis was performed using JMP Pro, version 12 (SAS Institute Inc., Cary, NC). All bivariate continuous correlations were performed using standard linear regression. One-way comparison of means was performed using the Student’s t-test, and one-tailed p-values are reported. Standard ANOVA was used for the comparison of means between more than two variables. MANOVA was used to assess changes between means of two groups over time. Generalized Linear Models were used to test the predictive nature of two or more continuous or categorical variables against a single continuous outcome. Kaplan-Meier survival curves and Cox proportional hazards models were performed using an endpoint defined as a single CD4+ T cell count reading less than 300 cells/μl, unless otherwise specified, and statistics reported for survival analyses are generated from the log-rank test.

## Supporting information

S1 Fig Effects of HLA class I and class II allele scores on CD4+ T cell decline. HLA class I score consist of the following alleles: HLA-B*1401, B*57, B*5801, and B*81. HLA class II scores consist of the following alleles: HLA-DQB1*02 and HLA-DRB1*15. Scores were calculated by summing the number of CD4-protective HLA class I or class II alleles for each individual for panel A and B, respectively. (A and B) Kaplan-Meier survival curves, with an endpoint defined as CD4 counts < 300/μl, demonstrating the effect of carriage of increasing numbers of protective HLA alleles on CD4+ T cell decline. P-values represent the comparison of decline trajectories between adjacent groups and were generated from a Cox proportional hazards model.
(DOCX)

S2 Fig Univariable comparisons between protective HLA alleles and set point viral load. HLA alleles initially defined as being protective for CD4+ T cell decline were evaluated for differences in mean set point viral loads between those carrying the allele (light blue bars) and those not carrying the allele (gray bars). Statistics based on individual *t* tests. ns (not significant); * p < 0.05; ** p < 0.01; *** p < 0.001.
(DOCX)

S3 Fig Alcohol consumption is an independent predictor of plasma LPS levels near seroconversion. Alcohol consumption data was collected through a revised version of the Alcohol Use Disorders Identification Test (AUDIT) questionnaire for couples in the ZEHRP cohort. Individuals with alcohol consumption data were divided into two groups, those that reported getting drunk monthly or more than monthly in the last year (n = 40), and those that reported getting drunk less than monthly or never in the last year (n = 58). (A) The graph depicts the comparison of the mean LPS levels early after HIV infection between these two groups. Statistics based on the Student’s *t* test, two-tailed p-value. (B) The table depicts a generalized linear model with protective HLA class I alleles and excessive alcohol consumption as predictors of plasma LPS levels near seroconversion.
(DOCX)

S4 Fig SNPs in linkage disequilibrium with four protective HLA class I alleles. **(A–D)** Graphs depict the linkage disequilibrium (LD) between individual SNPs within the MHC locus and four HLA class I alleles: B*1401, B*57, B*5801, and B*81. The r^2^ values for the linkage disequilibrium association between SNPs and each HLA class I allele are plotted on the y-axis. SNPs with r^2^ values >0.8 (above dotted line) are considered to be in strong LD.
(DOCX)

S1 Table Cohort characteristics. (DOCX)

S2 Table The effect of protective HLA alleles on CD4+ T cell decline is independent of set point viral load. (DOCX)

S3 Table Accelerated pathogenesis associated with increased LPS levels is independent of plasma viral loads at the time of LPS sampling. (DOCX)

S4 Table Associations between the levels of LPS and cellular immune activation are largely independent of plasma viremia at the time of PBMC collection. (DOCX)
