# Supplementary material for: Protective HLA alleles are associated with reduced LPS levels in acute HIV infection with implications for immune activation and pathogenesis
Source: PLoS Pathog. 2019 Aug 26;15(8):e1007981. doi: 10.1371/journal.ppat.1007981 (PMC6730937; doi:10.1371/journal.ppat.1007981)
Supplement: S1 Fig — HLA class I score consist of the following alleles: HLA-B*1401, B*57, B*5801, and B*81. HLA class II scores consist of the following alleles: HLA-DQB1*02 and HLA-DRB1*15. Scores were calculated by summing the number of CD4-protective HLA class I or class II alleles for each individual for panel A and B, respectively. (A and B) Kaplan-Meier survival curves, with an endpoint defined as CD4 counts < 300/μl, demonstrating the effect of carriage of increasing numbers of protective HLA alleles on CD4+ T cell decline. P-values represent the comparison of decline trajectories between adjacent groups and were generated from a Cox proportional hazards model. (DOCX) [file ppat.1007981.s001.docx]

**
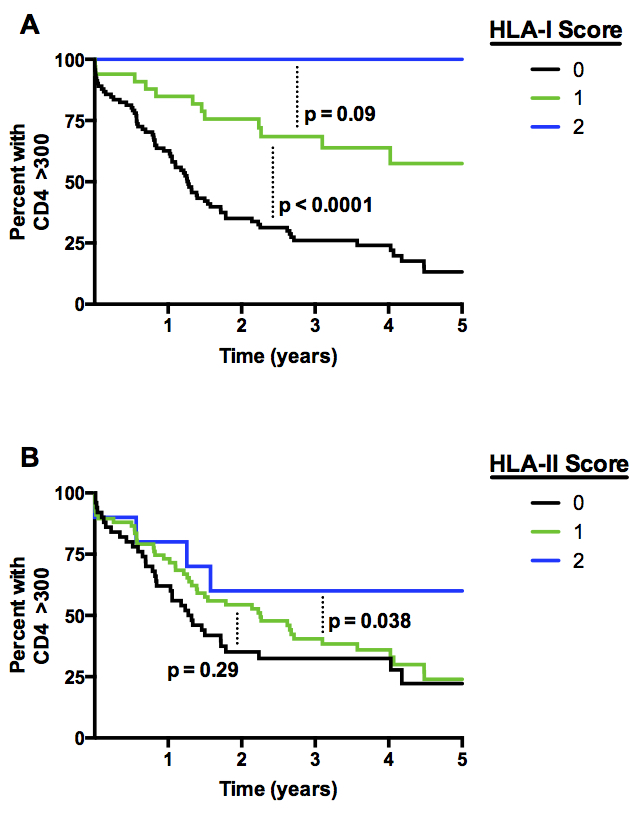
**

**S1 Fig. Effects of HLA class I and class II allele scores on CD4+ T cell decline.**HLA class I score consist of the following alleles: HLA-B*1401, B*57, B*5801, and B*81. HLA class II scores consist of the following alleles: HLA-DQB1*02 and HLA-DRB1*15. Scores were calculated by summing the number of CD4-protective HLA class I or class II alleles for each individual for panel A and B, respectively. (A and B) Kaplan-Meier survival curves, with an endpoint defined as CD4 counts < 300/µl, demonstrating the effect of carriage of increasing numbers of protective HLA alleles on CD4+ T cell decline. P-values represent the comparison of decline trajectories between adjacent groups and were generated from a Cox proportional hazards model.
