# Supplementary material for: Protective HLA alleles are associated with reduced LPS levels in acute HIV infection with implications for immune activation and pathogenesis
Source: PLoS Pathog. 2019 Aug 26;15(8):e1007981. doi: 10.1371/journal.ppat.1007981 (PMC6730937; doi:10.1371/journal.ppat.1007981)
Supplement: S2 Fig — HLA alleles initially defined as being protective for CD4+ T cell decline were evaluated for differences in mean set point viral loads between those carrying the allele (light blue bars) and those not carrying the allele (gray bars). Statistics based on individual t tests. ns (not significant); * p < 0.05; ** p < 0.01; *** p < 0.001. (DOCX) [file ppat.1007981.s002.docx]

**
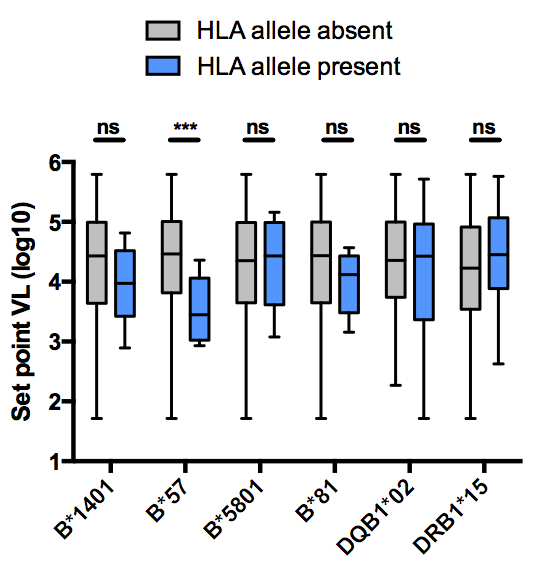


S2 Fig. Univariable comparisons between protective HLA alleles and set point viral load.** HLA alleles initially defined as being protective for CD4+ T cell decline were evaluated for differences in mean set point viral loads between those carrying the allele (light blue bars) and those not carrying the allele (gray bars). Statistics based on individual *t* tests. ns (not significant); * p < 0.05; ** p < 0.01; *** p < 0.001
