# Supplementary material for: Protective HLA alleles are associated with reduced LPS levels in acute HIV infection with implications for immune activation and pathogenesis
Source: PLoS Pathog. 2019 Aug 26;15(8):e1007981. doi: 10.1371/journal.ppat.1007981 (PMC6730937; doi:10.1371/journal.ppat.1007981)
Supplement: S3 Fig — Alcohol consumption data was collected through a revised version of the Alcohol Use Disorders Identification Test (AUDIT) questionnaire for couples in the ZEHRP cohort. Individuals with alcohol consumption data were divided into two groups, those that reported getting drunk monthly or more than monthly in the last year (n = 40), and those that reported getting drunk less than monthly or never in the last year (n = 58). (A) The graph depicts the comparison of the mean LPS levels early after HIV infection between these two groups. Statistics based on the Student’s t test, two-tailed p-value. (B) The table depicts a generalized linear model with protective HLA class I alleles and excessive alcohol consumption as predictors of plasma LPS levels near seroconversion. (DOCX) [file ppat.1007981.s003.docx]

**S3 Fig. Alcohol consumption is an independent predictor of plasma LPS levels near seroconversion.** Alcohol consumption data was collected through a revised version of the Alcohol Use Disorders Identification Test (AUDIT) questionnaire for couples in the ZEHRP cohort. Individuals with alcohol consumption data were divided into two groups, those that reported getting drunk monthly or more than monthly in the last year (n=40), and those that reported getting drunk less than monthly or never in the last year (n=58). (A) The graph depicts the comparison of the mean LPS levels early after HIV infection between these two groups. Statistics based on the Student’s *t* test, two-tailed p-value. (B) The table depicts a generalized linear model with protective HLA class I alleles and excessive alcohol consumption as predictors of plasma LPS levels near seroconversion.
