# Supplementary material for: Protective HLA alleles are associated with reduced LPS levels in acute HIV infection with implications for immune activation and pathogenesis
Source: PLoS Pathog. 2019 Aug 26;15(8):e1007981. doi: 10.1371/journal.ppat.1007981 (PMC6730937; doi:10.1371/journal.ppat.1007981)
Supplement: S4 Fig — (A–D) Graphs depict the linkage disequilibrium (LD) between individual SNPs within the MHC locus and four HLA class I alleles: B*1401, B*57, B*5801, and B*81. The r2 values for the linkage disequilibrium association between SNPs and each HLA class I allele are plotted on the y-axis. SNPs with r2 values >0.8 (above dotted line) are considered to be in strong LD. (DOCX) [file ppat.1007981.s004.docx]

**S4 Fig. SNPs in linkage disequilibrium with four protective HLA class I alleles.**

**(A – D)** Graphs depict the linkage disequilibrium (LD) between individual SNPs within the MHC locus and four HLA class I alleles: B*1401, B*57, B*5801, and B*81. The r^2^ values for the linkage disequilibrium association between SNPs and each HLA class I allele are plotted on the y-axis. SNPs with r^2^ values >0.8 (above dotted line) are considered to be in strong LD.
