# Supplementary material for: Protective HLA alleles are associated with reduced LPS levels in acute HIV infection with implications for immune activation and pathogenesis
Source: PLoS Pathog. 2019 Aug 26;15(8):e1007981. doi: 10.1371/journal.ppat.1007981 (PMC6730937; doi:10.1371/journal.ppat.1007981)
Supplement: S1 Table — (DOCX) [file ppat.1007981.s005.docx]

**S1 Table. Cohort characteristics.**

| Characteristic | Median (IQR)^a^ |
| --- | --- |
| Number of subjects | 127 |
| Number of females | 55 |
| Number of males | 72 |
| Age at seroconversion | 32 (29–38) |
| Days post EDI^b^ of first sample | 44 (33–49) |
| Set point viral load^c^ | 4.43 (3.67–4.99) |
| CD4 counts/μl at 1 year post infection | 398 (331–532) |

^a^Interquartile range (IQR) is defined as the 25^th^ to the 75^th^ percentiles

^b^Estimated date of infection

^c^Set point viral loads are defined as the log_10_-transformed copies of HIV RNA per mL of plasma and represent the nadir viral load value between 3 and 9 months post-infection for which subsequent values remain relatively stable
