# Supplementary material for: Protective HLA alleles are associated with reduced LPS levels in acute HIV infection with implications for immune activation and pathogenesis
Source: PLoS Pathog. 2019 Aug 26;15(8):e1007981. doi: 10.1371/journal.ppat.1007981 (PMC6730937; doi:10.1371/journal.ppat.1007981)
Supplement: S2 Table — (DOCX) [file ppat.1007981.s006.docx]

**S2 Table. The effect of protective HLA alleles on CD4+ T cell decline is independent of set point viral load.**

|  | **Cox Proportional Hazards Model (Time to CD4 counts < 300)** | | |
| --- | --- | --- | --- |
| **Factors Tested** | **HR** | **95% CI** | ***P*-value** |
| Female | .86 | .52–1.38 | .530 |
| Low vRC^a^ | .45 | .25–.77 | .003 |
| B*1401 | .18 | .01–.84 | .020 |
| B*57/5801 | .46 | .23–.84 | .010 |
| B*81 | <.01 | .00–.18 | <.0001 |
| DQB1*02 | .52 | .31–.83 | .006 |
| DRB1*15 | .44 | .26–.72 | .001 |
| Set point VL^b^ | 1.67 | 1.20–2.34 | .002 |

^a^Low viral replication capacity is defined as individuals infected with viruses falling within the lowest tercile the range tested

^b^Set point viral loads are defined as the log10-transformed copies of HIV RNA per mL of plasma and represent the nadir viral load value between 3 and 9 months post-infection for which subsequent value remain relatively stable

HR, hazard ratio as risk per unit change in regressor; CI, confidence interval; vRC, viral replication capacity; VL, viral load
