# Supplementary material for: Protective HLA alleles are associated with reduced LPS levels in acute HIV infection with implications for immune activation and pathogenesis
Source: PLoS Pathog. 2019 Aug 26;15(8):e1007981. doi: 10.1371/journal.ppat.1007981 (PMC6730937; doi:10.1371/journal.ppat.1007981)
Supplement: S3 Table — (DOCX) [file ppat.1007981.s007.docx]

**S3 Table. Accelerated pathogenesis associated with increased LPS levels is independent of plasma viral loads at the time of LPS sampling.**

|  | **Cox Proportional Hazards Model (Time to CD4 counts < 300)** | | |
| --- | --- | --- | --- |
| **Factors Tested** | **HR^a^** | **95% CI** | ***P*-value** |
| LPS at seroconversion (log10) | 3.15 | 1.35–8.21 | .006 |
| VL at seroconversion (log10) | 1.45 | 1.13–1.89 | .003 |

^a^Hazard Ratio is expressed as per unit change in regressor
