# Supplementary material for: Protective HLA alleles are associated with reduced LPS levels in acute HIV infection with implications for immune activation and pathogenesis
Source: PLoS Pathog. 2019 Aug 26;15(8):e1007981. doi: 10.1371/journal.ppat.1007981 (PMC6730937; doi:10.1371/journal.ppat.1007981)
Supplement: S4 Table — (DOCX) [file ppat.1007981.s008.docx]

**S4 Table. Associations between the levels of LPS and cellular immune activation are largely independent of plasma viremia at the time of PBMC collection.**

|  | **Generalized Linear Model**  **(LPS and pVL as predictors^a^)** | | | | | |
| --- | --- | --- | --- | --- | --- | --- |
|  | **Post-infection LPS** | | | **Log_10_ VL at PBMC sampling** | | |
| Outcome Tested | β | Conf. Limit | p-value | β | Conf. Limit | p-value |
| CD4+ T_CM_ PD-1+ | 0.57 | 0.01 – 1.12 | 0.045 | 0.82 | -2.04 – 3.68 | 0.55 |
| CD4+ T_EM_ PD-1+ | 1.02 | 0.13 – 1.91 | 0.027 | 2.38 | -2.21 – 6.98 | 0.29 |
| CD8+ CD38 MFI | 26.8 | -2.77 – -0.34 | 0.07 | 128.3 | -24.3 – 281 | 0.09 |
| CD8+ CD38+/HLA-DR+ | 0.90 | 0.41 – 1.38 | 0.001 | 1.77 | -0.73 – 4.28 | 0.16 |
| CD8+ Ki67+ | 0.38 | 0.16 – 0.61 | 0.002 | 1.57 | 0.39 – 2.75 | 0.01 |

^a^Generalized Linear Model (GLM) where LPS levels and plasma viral load at the time of LPS sampling were used as predictors for the T cell activation phenotypes evaluated in the far left column
